# Noninvasive ventilation for severely acidotic patients in respiratory intermediate care units: Precision medicine in intermediate care units

**DOI:** 10.1186/s12890-016-0262-9

**Published:** 2016-07-07

**Authors:** Juan F. Masa, Isabel Utrabo, Javier Gomez de Terreros, Myriam Aburto, Cristóbal Esteban, Enric Prats, Belén Núñez, Ángel Ortega-González, Luis Jara-Palomares, M. Jesus Martin-Vicente, Eva Farrero, Alicia Binimelis, Ernest Sala, José C. Serrano-Rebollo, Emilia Barrot, Raquel Sánchez-Oro-Gomez, Ramón Fernández-Álvarez, Francisco Rodríguez-Jerez, Javier Sayas, Pedro Benavides, Raquel Català, Francisco J. Rivas, Carlos J. Egea, Antonio Antón, Patricia Peñacoba, Ana Santiago-Recuerda, M. A. Gómez-Mendieta, Lidia Méndez, José J. Cebrian, Juan A. Piña, Enrique Zamora, Gonzalo Segrelles

**Affiliations:** San Pedro de Alcántara Hospital, C/Rafael Alberti 12, 10005 Cáceres, Spain; Galdakao-Usansolo Hospital, Bilbao, Spain; Belvitge Hospital, Barcelona, Spain; Son Espases Hospital, Palma de Mallorca, Spain; Nuestra Señora del Prado Hospital, Talavera de la Reina, Toledo Spain; Virgen del Rocío Hospital, Sevilla, Spain; Central de Asturias Hospital, Oviedo, Spain; Doce de Octubre Hospital, Madrid, Spain; “Sant Joan” University Hospital, Universitat Rovira i Virgili, IISPV, Reus, Tarragona Spain; Txaguritxu Hospital, Vitoria, Spain; Sant Pau Hospital, Barcelona, Spain; La Paz Hospital, Madrid, Spain; Universitario Lucus Augusti Hospital, Lugo, Spain; Costa del Sol Hospital, Málaga, Spain; La Princesa Hospital, Madrid, Spain; CIBER de Enfermedades Respiratorias (CIBERES), Madrid, Spain

**Keywords:** Noninvasive ventilation, Respiratory intermediate care unit, Acute hypercapnic respiratory failure, COPD, Acute pulmonary edema, Obesity hypoventilation syndrome

## Abstract

**Background:**

Severe acidosis can cause noninvasive ventilation (NIV) failure in chronic obstructive pulmonary disease (COPD) patients with acute hypercapnic respiratory failure (AHRF). NIV is therefore contraindicated outside of intensive care units (ICUs) in these patients. Less is known about NIV failure in patients with acute cardiogenic pulmonary edema (ACPE) and obesity hypoventilation syndrome (OHS). Therefore, the objective of the present study was to compare NIV failure rates between patients with severe and non-severe acidosis admitted to a respiratory intermediate care unit (RICU) with AHRF resulting from ACPE, COPD or OHS.

**Methods:**

We prospectively included acidotic patients admitted to seven RICUs, where they were provided NIV as an initial ventilatory support measure. The clinical characteristics, pH evolutions, hospitalization or RICU stay durations and NIV failure rates were compared between patients with a pH ≥ 7.25 and a pH < 7.25. Logistic regression analysis was performed to determine the independent risk factors contributing to NIV failure.

**Results:**

We included 969 patients (240 with ACPE, 540 with COPD and 189 with OHS). The baseline rates of severe acidosis were similar among the groups (45 % in the ACPE group, 41 % in the COPD group, and 38 % in the OHS group). Most of the patients with severe acidosis had increased disease severity compared with those with non-severe acidosis: the APACHE II scores were 21 ± 7.2 and 19 ± 5.8 for the ACPE patients (*p* < 0.05), 20 ± 5.7 and 19 ± 5.1 for the COPD patients (*p* < 0.01) and 18 ± 5.9 and 17 ± 4.7 for the OHS patients, respectively (NS). The patients with severe acidosis also exhibited worse arterial blood gas parameters: the PaCO_2_ levels were 87 ± 22 and 70 ± 15 in the ACPE patients (*p* < 0.001), 87 ± 21 and 76 ± 14 in the COPD patients, and 83 ± 17 and 74 ± 14 in the OHS patients (NS)., respectively Further, the patients with severe acidosis required a longer duration to achieve pH normalization than those with non-severe acidosis (patients with a normalized pH after the first hour: ACPE, 8 % vs. 43 %, *p* < 0.001; COPD, 11 % vs. 43 %, *p* < 0.001; and OHS, 13 % vs. 51 %, *p* < 0.001), and they had longer RICU stays, particularly those in the COPD group (ACPE, 4 ± 3.1 vs. 3.6 ± 2.5, NS; COPD, 5.1 ± 3 vs. 3.6 ± 2.1, *p* < 0.001; and OHS, 4.3 ± 2.6 vs. 3.7 ± 3.2, NS). The NIV failure rates were similar between the patients with severe and non-severe acidosis in the three disease groups (ACPE, 16 % vs. 12 %; COPD, 7 % vs. 7 %; and OHS, 11 % vs. 4 %). No common predictive factor for NIV failure was identified among the groups.

**Conclusions:**

ACPE, COPD and OHS patients with AHRF and severe acidosis (pH ≤ 7.25) who are admitted to an RICU can be successfully treated with NIV in these units. These results may be used to determine precise RICU admission criteria.

**Electronic supplementary material:**

The online version of this article (doi:10.1186/s12890-016-0262-9) contains supplementary material, which is available to authorized users.

## Background

Noninvasive ventilation (NIV) is a standard treatment for acute hypercapnic respiratory failure (AHRF) in chronic obstructive pulmonary disease (COPD) [1, 2] because it avoids oro-tracheal intubation (OTI) and the mortality associated with this procedure.

The use of NIV in obesity hypoventilation syndrome (OHS) with AHRF is increasing as a result of the worldwide obesity epidemic, although evidence regarding its use is currently limited to clinical series and observational studies [3]. The efficacy of NIV and outcomes appear to be similar between these patients and COPD patients [3].

NIV and continuous positive airway pressure (CPAP) improve both the symptoms and physiological variables in patients with acute cardiogenic pulmonary edema (ACPE), [4–6] although it is not clear whether either intervention results in a lower mortality rate than standard treatments [5, 7, 8].

Currently, NIV for AHRF is primarily used to treat three diseases, namely COPD, [3, 9–11] OHS, [3, 12] and ACPE, [4, 6, 11] which are associated with non-negligible rates of NIV failure. Severe acidosis is an important prognostic factor in AHRF patients [13, 14]. Different studies have examined factors that may be predictive of NIV treatment failure in AHRF. In COPD patients, a baseline acidic pH (i.e., <7.25) explains many cases of failure, [11, 15–21] and this finding prompted the publication of different guidelines and reviews [22, 23] that do not recommend NIV outside of the intensive care unit (ICU) in patients with a baseline pH of <7.25. There is no uniform standard regarding NIV for ACPE, [24, 25] and no information is available on NIV for OHS.

NIV is routinely administered in specific units outside of the ICU, including step-down units, high-dependency units, emergency departments, and respiratory intermediate care units (RICUs). RICUs have grown in number because they allow for NIV to be performed on patients with less severe illness, with similar results as those reported in ICUs but at a lower cost [26]. Patients admitted to these units are frequently hemodynamically stable and without multiple organ failure, and they often have a do-not-intubate (DNI) order [26, 27]. Currently, insufficient information is available to determine whether the same factors that predict NIV failure in ICU patients are applicable to RICU patients and whether a pH of <7.25 should be considered a contraindication to NIV in these patients.

Although the use of intermediate care units is growing, [28] more precision is needed when determining the criteria for admitting patients to these units. Because the characteristics of patients admitted to RICUs and ICUs differ and because baseline severe acidosis is recognized as a poor prognostic indicator in ICU patients, the primary objective of this prospective multicenter study was to evaluate NIV failure rates in patients admitted to an RICU with AHRF resulting from ACPE, COPD, or OHS and either severe or non-severe acidosis (pH ≤ 7.25 or pH > 7.25, respectively). The secondary objectives included the following: 1) to compare baseline characteristics and improvements in patients with and without severe acidosis; 2) to compare RICU and hospitalization durations between the groups of patients; and 3) to identify the factors that predict NIV treatment failure in ACPE, COPD, and OHS.

## Methods

This prospective observational study was conducted in the seven RICUs that participated in the cohort developed by the Spanish RICU group.

### Patients

AHRF patients with ACPE, COPD, or OHS who were consecutively admitted between 2011 and 2013 and who received NIV as an initial ventilatory support measure were prospectively included in this study. AHRF was defined as worsening dyspnea and a pH of ≤7.35 with a PCO_2_ of ≥45 mmHg. We excluded patients with the following characteristics/conditions: 1) urgent necessity for OTI in an emergency department; 2) persistent hemodynamic instability (except in patients with a DNI order); 3) multiorgan failure (except in patients with a DNI order); 4) COPD without forced spirometry (completed either before or after AHRF); 5) pneumonia (except in patients with a DNI order); 6) acute myocardial infarction (except in patients with a DNI order); and 7) deep hypercapnic coma preventing NIV. The ethics committee of the CEIC San Pedro de Alcantara Hospital approved this study, and informed consent was obtained from all participants.

### RICU characteristics

All of the RICUs in our consortium were located at a dedicated site within the hospital that was either integrated with or adjacent to a pulmonary area and that had access to noninvasive monitoring procedures (including electrocardiogram, transcutaneous PCO_2_, oxygen saturation, blood pressure, and respiratory rate monitoring and the monitoring of basic curves from ventilators); in addition, they had a nurse-patient ratio of 1/4–6 nurses who were specially trained in NIV and 24-h physician coverage. All of the facilities except for two had 4 beds and admitted a mean of 320 RICU patients per year. The inhabitants per RICU ranged from 500,000–1,500,000 among the facilities. Each RICU had an experienced pulmonologist who was available for morning rounds. Approximately half of the units provided the same specialized care during evenings, nights, and weekends, whereas the other half were attended by ICU specialists during these times. In Spain, pulmonology and intensive care are different specialties with different services. The models and brands of ventilators used in the RICUs are described in Additional file 1.

### Protocol

Baseline arterial blood gases were measured at approximately one hour after standard treatment. Pressure support with bi-level pressure (PSV) was initially used in all patients, although the specialists were able to transition to volume-targeted PSV, pressure control ventilation, or volume control ventilation, depending on the evolution of the patient. Oxygen was added to maintain the desired SpO_2_ (i.e., ≥92 %). Preliminary ventilatory adjustments were initiated based on both efficacy and patient tolerance. A priori, the highest expiratory pressure was set for OHS (i.e., 9 cm H_2_O), an intermediate pressure was set for ACPE (i.e., 6 cm H_2_O), and the lowest pressure was set for COPD (i.e., 4 cm H_2_O). Final ventilatory adjustments were performed after accounting for basic curves (flow, pressure, and volume) and arterial blood gases. After NIV was initiated, arterial blood gas samples were extracted at baseline, after one hour, after 24 h and at the time of discharge from the RICU. Either oro-nasal devices or full facemasks were initially used, although the physicians were allowed to transition to a nasal mask or full mask if necessary. In our consortium, the duration of NIV was based on the maintenance of a stable pH of ≥7.35, with breaks allowed for eating as tolerated by the patient. ACPE and COPD patients received standard medical treatment. If necessary, OHS patients received additional treatment with bronchodilators, antibiotics, anti-arrhythmics, or diuretics.

### Definitions

ACPE was defined based on both clinical and radiological criteria. COPD exacerbations were defined using clinical criteria, and a diagnosis of COPD was made based on clinical and spirometric criteria (a forced expiratory volume in one second (FEV1) of <80 % of the predicted volume and an FEV1/forced vital capacity (FVC) of <70 %) [29]. OHS was defined as a body mass index (BMI) of ≥30 and a baseline PCO_2_ of ≥45 mmHg in the absence of a current or previous medical history of another potential disease that could cause hypercapnia, including lung, metabolic, neuromuscular, and chest wall diseases.

NIV treatment failure was diagnosed when one of the following occurred during a patient’s hospitalization: a) OTI was performed in the RICU due to predefined criteria (see Additional file 1); b) the patient was transferred to an ICU for OTI (see Additional file 1) and conventional invasive ventilation, multiple organ failure, or refractory hypotension (“a” and “b” only for patients without a DNI order); or c) the patient died.

A type 1 RICU was defined as an RICU with 24-h coverage by pulmonologists. A type 2 RICU was defined as an RICU with 24-h coverage split between pulmonologists in the morning and ICU specialists for the remainder of the day and over the weekend.

### Variables

The primary variable examined in this study was the NIV treatment failure rate. We also evaluated the following secondary variables: age, gender, BMI, smoking status, alcohol consumption, respiratory rate, systolic and diastolic blood pressures, comorbidities according to the Charlson index, [30] disease severity according to APACHE II scores, arterial blood gas parameters (PO_2_, PCO_2_, and pH), PO_2_/FiO_2_ rate, spirometry (FEV1 and FVC) in the COPD group, RICU type, ventilator mode, previous treatment with either CPAP or NIV (at home or in the hospital), and the presence of a DNI order.

### Statistical analysis

We used either the *t*-test (for normally distributed data) or the Mann-Whitney *U* test (for non-normally distributed data) for continuous variables and the *χ*2 test for categorical variables for comparisons of the results among all samples or for comparisons of the results for each of the three groups (ACPE, COPD, and OHS) between the patients with and without severe acidosis. The following parameters were analyzed: 1) baseline values; 2) changes in arterial blood gas parameters (at baseline and discharge); 3) percentage of patients with a pH ≥7.35 after one hour, after 24 h, and at the time of discharge (to assess the RICU courses of the patients); 4) hospitalization and RICU durations (days); and 5) incidence of NIV treatment failure. Comparisons 3, 4 and 5 were repeated for each of the DNI sub-groups (without and with a DNI order). Missing values due to NIV treatment failure were replaced with the last available value. Finally, we performed logistic regression analysis using the variables with a *p* < 0.2 in univariate analysis to identify predictive factors for NIV treatment failure in each disease group.

## Results

A total of 969 patients were included in this study (240 with ACPE, 540 with COPD, and 189 with OHS). Table 1 shows the baseline characteristics of all patients. The patients with ACPE were older, and those with COPD were more likely to be male and an active smoker. The mean pH and markers of disease severity and comorbidities were very similar among the groups.

**Table 1:** Baseline characteristics

	ACPE *N* = 240	COPD *N* = 540	OHS *N* = 189	All *N* = 969
Age, years, X (SD)	75 (13)	71 (9)	71 (11)	72 (10)
Sex, male, %	42	87	38	67
BMI, X (SD)	31 (6)	28 (6)	39 (8)	31 (8)
Current smoker, %	11	52	31	38
Current drinker, %	10	18	17	16
Respiratory rate, X (SD)	25.4 (4.1)	25.7 (4.6)	23.9 (4)	25 (5)
Systolic BP, mmHg, X (SD)	134 (21)	134 (20)	134 (19)	134 (20)
Diastolic BP, mmHg, X (SD)	72 (15)	73 (11)	72 (11)	73 (13)
Charlson index, X (SD)	2.8 (1.2)	2.3 (1.1)	2 (1.1)	2.4 (1.2)
Glasgow scale, X (SD)	13.6 (1.8)	13.9 (1.1)	13.8 (1.6)	13.8 (1.4)
APACHE II score, X (SD)	19.8 (6.5)	19.1 (5.3)	18.1 (5.8)	19 (5.7)
PaO_2_, mmHg, X (SD)	67 (30)	62 (28)	59 (21)	63 (28)
PaO_2_/FiO_2_, X (SD)	185 (91)	191 (89)	178 (74)	187 (87)
PaCO_2_, mmHg, X (SD)	78 (20)	81 (18)	77 (15)	79 (18)
pH, X (SD)	7.25 (0.08)	7.25 (0.07)	7.26 (0.07)	7.25 (0.07)
FVC, %, X (SD)		61 (12)		
FEV1, %, X (SD)		39 (10)		
RICU type 1, %	66	60	49	59
PS mode, %	74	83	68	78
CPAP/NIV, %	17	21	31	22
DNI order, %	65	66	54	63

*Abbreviations*: *X (SD)* mean and standard deviation, *BMI* body mass index, *BP* blood pressure, *RICU* respiratory intermediate care unit, *PS* pressure support, *CPAP/NIV* previous continuous positive airway pressure or noninvasive ventilation, *DNI* do not intubate

Table 2 presents the baseline variables related to severe and non-severe acidosis. The proportions of patients with severe acidosis were similar among the groups (45 % in the ACPE group, 41 % in the COPD group, and 38 % in the OHS group). Interestingly, the BMIs of the COPD and OHS patients with severe acidosis were decreased. Active smokers were less likely to have COPD with severe acidosis. The patients with OHS and severe acidosis had higher systolic and diastolic blood pressures, while those with ACPE and severe acidosis suffered from a larger number of comorbidities. Further, the patients with severe acidosis, especially those with ACPE, were more likely to receive care in a type 1 RICU.

**Table 2:** Baseline variables for both severe and non-severe acidosis

	ACPE *N* = 240		COPD *N* = 540		OHS *N* = 189		All *N* = 969	
pH Patients	≤7.25 109	>7.25 131	≤7.25 219	>7.25 322	≤7.25 72	>7.25 117	≤7.25 399	>7.25 570
Age, years, X (SD)	76 (10)	75 (10)	71 (9)	70 (9)	72 (10)	70 (11)	73 (10)	71 (10)^**^
Sex, male, %	40	44	88	86	49	29^**^	61	46^***^
BMI, X (SD)	33 (7)	30 (8)^*^	26 (5)	29 (6)^***^	37 (8)	40 (8)^**^	30 (7)	32 (8)^**^
Smoker, %	6.3	4.8	16	36^***^	11	19	12	23^**^
Drinker, %	3.2	6.5	6	12^*^	9	9	5	9^*^
Respiratory rate, X (SD)	25.4 (6.6)	25.3 (4.5)	25.8 (3.9)	25.6 (5.1)	24.6 (3.7)	23.5 (4.1)^*^	25.5 (3.8)	25.1 (4.8)^*^
Systolic BP, mmHg, X (SD)	133 (24)	135 (19)	133 (20)	134 (21)	143 (20)	129 (17)^***^	135 (21.1)	133.3 (19.8)
Diastolic BP, mmHg, X (SD)	72 (17)	73 (14)	72 (10)	74 (12)^*^	76 (10)	69 (11)^***^	72.6 (12.2)	72.5 (12.5)
Charlson index, X (SD)	3.2 (1.3)	2.5 (1)^***^	2.2 (1.2)	2.3 (1.1)	2.2 (1.5)	1.9 (0.9)	2.5 (1.4)	2.3 (1.1)^*^
Glasgow scale, X (SD)	13.4 (1.6)	13.7 (1.9)^***^	13.7 (1.1)	14 (1.1)^***^	13.5 (1.9)	14 (1.3)^**^	13.6 (1.4)	14 (1.4)^***^
APACHE II score, X (SD)	20.7 (7.2)	19.1 (5.8)^*^	19.7 (5.7)	18.7 (5.1)^**^	18.3 (5.9)	17.4 (4.7)	19.7 (6.2)	18.5 (5.2)^**^
PaO_2_, mmHg, X (SD)	70 (28)	65 (32)^*^	64 (35)	61 (22)	59 (17)	59 (24)	65 (31)	61 (25)
PaO_2_/FiO_2_, X (SD)	178 (82)	191 (97)	180 (103)	198 (76)^***^	164 (62)	186 (80)^*^	176 (92)	194 (82)^***^
PaCO_2_, mmHg, X (SD)	87 (22)	70 (15)^***^	87 (21)	76 (14)^***^	83 (17)	74 (14)^**^	86 (21)	75 (14)^***^
PH, X (SD)	7.18 (0.07)	7.30 (0.03)^***^	7.19 (0.05)	7.30 (0.25)^***^	7.19 (0.05)	7.30 (0.03)^***^	7.18 (0.06)	7.30 (0.03)^***^
FVC, %, X (SD)			60 (10)	62 (12)^*^				
FEV1, %, X (SD)			36 (9)	41 (10)^***^				
RICU type 1, %	78	57^**^	62	59	58	43	66	55^**^
PS mode, %	67	69	72	88^*^	50	69^*^	68	81^**^
CPAP/NIV, %	11	22	17	24	38	27	19	24
DNI order, %	66	64	75	59^***^	56	53	68	59^**^

*Abbreviations*: *X (SD)* mean (standard deviation), *BMI* body mass index, *BP* blood pressure, *RICU* respiratory intermediate care unit, *PS* pressure support, *CPAP/NIV* previous continuous positive airway pressure or noninvasive ventilation, *DNI* do not intubate

^*^ *P* < 0.05, ^**^ *P* < 0.01, and ^***^ *P* < 0.001

PSV was less frequently used by OHS patients with severe acidosis and COPD; instead, other modes of ventilation, particularly PSV plus target volume, were used (see Additional file 1: Figure S1). Less than 5 % of the patients were treated with heated humidification or used masks other than oro-nasal masks.

**Fig. 1:**
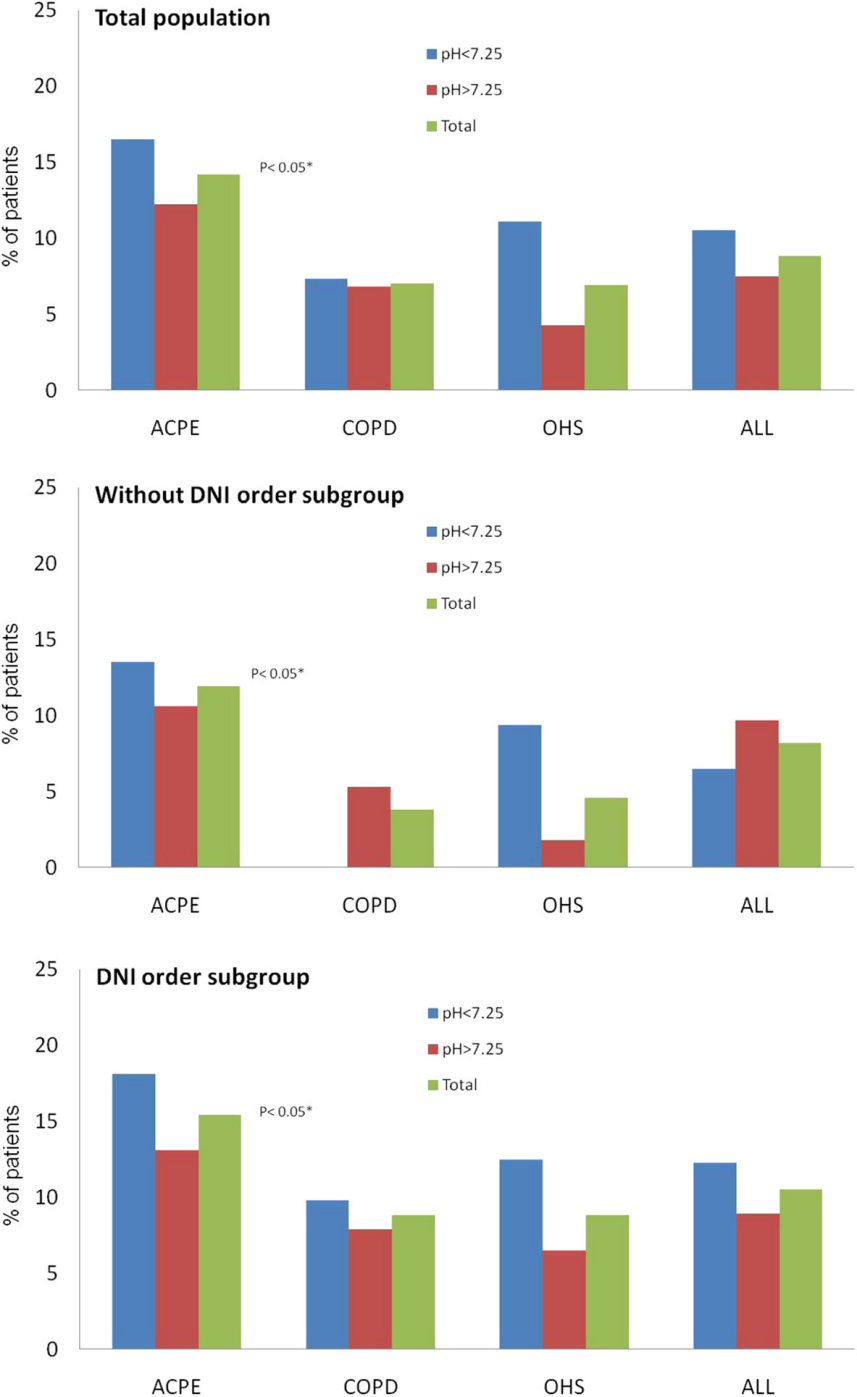
The percentages of patients experiencing NIV treatment failure during hospitalization are shown for the patients with severe and non-severe acidosis, for the whole sample population, and for the patients with each of the three diseases studied (ACPE, COPD, and OHS). Data are presented for the total population and for the sub-groups with and without a DNI order. Note that none of the COPD patients with a DNI order had a pH ≤7.25. Abbreviations: NIV = noninvasive ventilation, ACPE = acute cardiogenic pulmonary edema, COPD = chronic obstructive pulmonary disease, OHS = obesity hypoventilation syndrome, and DNI = do not intubate

No significant differences were observed in the NIV failure rate between the patients with severe and non-severe acidosis (Fig. 1). Further, similar results and statistical significance values were observed between the two DNI sub-groups, with the exception of the more frequent occurrence of NIV failure among the patients with COPD and severe acidosis who had a DNI order. Overall, the patients with a DNI order tended to experience NIV failure more frequently. Further, when all NIV failures were considered (e.g., the patients were not divided according to severe and non-severe acidosis), the ACPE patients had the highest NIV failure rate.

The data obtained from univariate analysis and the regression models used to analyze the NIV failure rates are shown in Table 3. In the ACPA group, old age and an increased respiratory rate were predictive of NIV failure, while in the COPD group, a low PaO_2_ and a pH < 7.35 within the first hour of NIV treatment were predictive factors. In the OHS group, only elevated systolic blood pressure was predictive of NIV failure. The presence of a DNI order was not a predictive factor.

**Table 3:** A regression model with NIV failure as the dependent variable

	ACPE		COPD		OHS	
	*P* value^*^	Final model ExpB (CI)	*P* value^*^	Final model ExpB (CI)	*P* value^*^	Final model ExpB (CI)
Age, years^a^	0.03	1.096 (1.035–1.161)	0.029	NS	0.104	NS
Male^b^	0.534		0.558		1	
BMI^a^	0.699		0.715		0.269	
Smoker^b^	1		0.552		1	
Drinker^b^	0.580		0.450		1	
Respiratory rate^a^	0.068	1.094 (1.002–1.194)	0.555		0.301	
SBP, mmHg^a^	0.325		0.972		0.001	1.040 (1.014–1.067)
DBP, mmHg^a^	0.724		0.385		0.337	
Charlson index^a^	0.880		0.824		0.585	
Glasgow scale^a^	0.267		0.334		0.159	NS
APACHE II score^a^	0.143		0.749		0.180	
PaO_2_, mmHg^a^	0.859		0.109	1.019 (1.000–1.019)	0.819	
PaO_2_/FiO_2_^a^	0.861		0.213		0.540	
PaCO_2_, mmHg^a^	0.781		0.119	NS	0.101	NS
pH ≤ 7.25^b^	0.359		0.865		0.083	NS
FVC %^a^			0.774			
FEV1 %^a^			0.472			
RICU type 1^b^	0.696		0.864		0.778	
PS mode^b^	1		1		0.316	
CPAP/NIV^b^	1		1		1	
DNI order^b^	0.562		0.033	NS	0.388	
pH ≥ 7.35 at 1 h^b^	0.027	NS	0.042	0.341 (0.117–0.999)	0.058	NS

*Abbreviations*: *ExpB* exponent B, *CI* confidence interval, *NS* not significant, *BMI* body mass index, *SBP* systolic blood pressure, *DBP* diastolic blood pressure, *RICU* respiratory intermediate care unit, *PS* pressure support, *CPAP/NIV* previous continuous positive airway pressure or noninvasive ventilation, *DNI* do not intubate

^a^continuous variables in the model, ^b^categorical variables in the model, and ^*^ *P* values from univariate analysis. Variables with a P ≤ 0.2 were included in regression analysis

Changes in the arterial blood gas parameters were detected between baseline and discharge in the patients with severe and non-severe acidosis who received NIV (Table 4). Improvement in the PaO_2_/FiO_2_ ratio was only detected in the OHS patients with severe acidosis. In addition, the patients with severe acidosis in all three groups exhibited improvements in PaCO_2_ and pH.

**Table 4:** Changes in arterial blood gas parameters between baseline and discharge following NIV treatment in patients with severe and non-severe acidosis

	APE *N* = 240		COPD *N* = 540		OHS *N* = 189		All *N* = 969	
pH	≤7.25	>7.25	≤7.25	>7.25	≤7.25	>7.25	≤7.25	>7.25
PaO_2_, mmHg, X (SD)	+2.9 (30)	−2.2 (32)	−4 (33)	−7.6 (36)	−10 (20)	−4.7 (26)	−3.3 (31)	−5.7 (33)
PaO_2_/FiO_2_, X (SD)	−90 (102)	−74 (103)	−66 (112)	−78 (139)	−135 (100)	−72 (97)^**^	−85 (110)	−75 (123)^*^
PaCO_2_, mmHg, X (SD)	+29 (22)	+13 (16)^**^	+26 (22)	+17 (15)^**^	+28 (18)	+15 (14)^**^	+27 (21)	+16 (15)^**^
pH, X (SD)	−0.21 (0.11)	−0.11 (0.07)^**^	−0.23 (0.08)	−0.12 (0.06)^**^	−0.22 (0.09)	−0.12 (0.05)^**^	−0.22 (0.09)	−0.12 (0.06)^**^

*Abbreviations*: *X (SD)* mean (standard deviation), *NIV* noninvasive ventilation

^*^ *P* < 0.01 and ^**^ *P* < 0.001

**Fig. 2:**
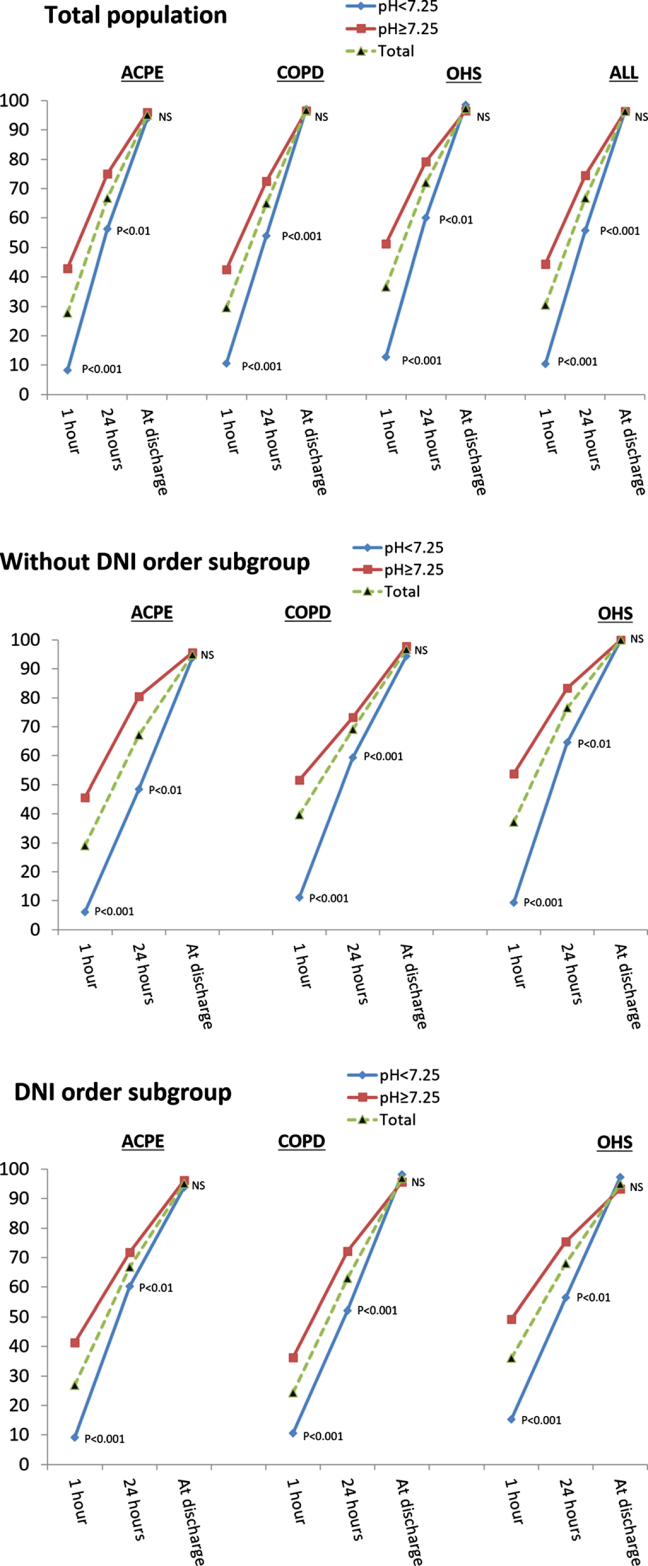
The percentages of patients with a normal pH (pH ≥ 7.35) after one hour, after 24 h, and at the time of discharge are shown for the patients with and without severe acidosis who presented with each of the three diseases studied (ACPE, COPD, and OHS) and for the total population. Data are presented for the total population and for the sub-groups with and without a DNI order. The *P* values indicate the significance of results obtained in comparisons of the percentages of patients with and without severe acidosis with a normal pH after one hour, after 24 h and at the time of RICU discharge. Abbreviations: ACPE = acute cardiogenic pulmonary edema, OHS = obesity hypoventilation syndrome, RICU = respiratory intermediate care unit, and DNI = do not intubate

The percentage of patients with or without acidosis and a pH ≥ 7.35 after 1 h, after 24 h, and at the time of discharge are shown in Fig. 2. The patients with severe acidosis in all three disease groups took longer to achieve pH normalization. The patients with OHS appeared to improve more rapidly than those suffering from the other two diseases. Similar results were obtained for both sub-groups (those with and without a DNI order), although a tendency toward requiring a longer duration to achieve pH normalization was observed in the patients with a DNI order.

**Fig. 3:**
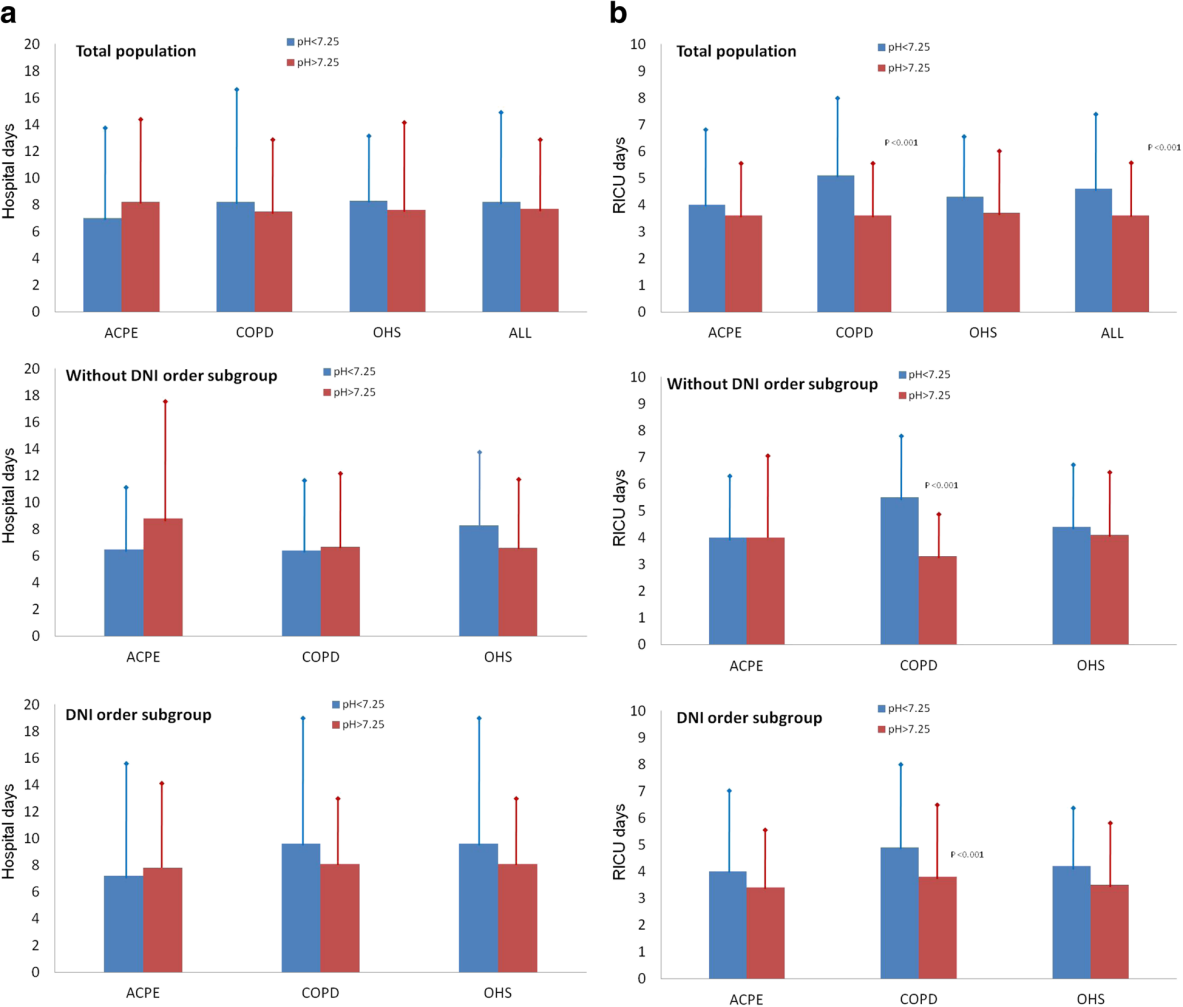
Comparison of the hospitalization (Panel **a**) and RICU stay (Panel **b**) durations between the patients with severe and non-severe acidosis. Data are presented for the total population and for the sub-groups with or without a DNI order. Abbreviations: RICU = respiratory intermediate care unit, ACPE = acute cardiogenic pulmonary edema, OHS = obesity hypoventilation syndrome, and DNI = do not intubate

The hospitalization and RICU durations were compared between the patients with a pH ≤ 7.25 and those with a pH > 7.25 (Fig. 3). While no differences were observed in the hospitalization duration (Fig. 3a), the duration of RICU stay was longer in the severe acidosis group. A significant difference in the duration of RICU stay was detected only in the COPD group (Fig. 3b). Similar results were obtained for both DNI sub-groups.

## Discussion

The primary results of this large observational study are as follows: 1) there was no significant difference in the NIV failure rate among the patients with severe and non-severe acidosis and any of the diseases studied. Hence, severe baseline acidosis was not an adjusted predictive factor for NIV failure in any of the examined diseases. 2) No common predictive factors were identified among the diseases by regression analysis. 3) The time required to achieve pH normalization was longer in the severe acidosis group, regardless of the disease. 4) Finally, there was no difference in hospitalization duration between the patients with severe and non-severe acidosis, regardless of the disease. However, the patients with COPD and severe acidosis remained in the RICU for a longer duration than those with COPD and non-severe acidosis.

The results of our study highlight several questions:

- Does baseline acidosis predict an unfavorable outcome in RICU patients? One previous study specifically compared NIV failure rates among 29 COPD patients with AHRF and severe (pH < 7.25) or non-severe (pH ≥ 7.25) baseline acidosis [9] and found no significant differences between the groups. The clinical and functional characteristics of the patients and the site at which NIV was performed were similar to those in the present study. Other studies have explored the predictive value of stratified baseline pH for NIV failure in COPD patients with AHRF [15, 16] and have revealed that severe acidosis at baseline is a predictive factor. Notably, Plant’s study [16] was performed without the monitoring capabilities of a special unit (i.e., an RICU); in addition, one of the exclusion criteria in that study was a pH < 7.25. Confalonieri’s study [15] was performed in the general wards, RICU, and ICU of a hospital. Therefore, differences in patient characteristics, severity (baseline pH values =7.32 for Plant’s study and 7.28 for Confalonieri’s study), and NIV application sites may explain the differing results of these studies. Finally, other studies conducted in ICUs have supported the predictive value of baseline pH without stratification [17, 20, 21] and have demonstrated that baseline acidosis is predictive of NIV failure. Although the baseline pH values used in these studies are similar to those used in our study, patients admitted to the ICU may have other negative prognostic factors, such as multiorgan failure or persistent hemodynamic instability, which may favor different outcomes.
  Data regarding NIV for ACPE are scarcer than those pertaining to NIV for COPD, particularly in patients with AHRF. Previous randomized trials performed in emergency departments [4, 31, 32] have compared NIV efficacy in non-hypercapnic and hypercapnic (with more severe acidosis) sub-groups and have demonstrated that hypercapnia is not predictive of NIV failure. Other studies have examined whether baseline acidosis is a predictor of unfavorable outcomes in patients with and without hypercapnia, although the mean PaCO_2_ was higher than 45 mmHg in these patients [24, 25, 33]. In two studies conducted in emergency departments [24, 33], baseline acidosis was not found to be a negative prognostic factor. However, it was found to be a negative prognostic factor in studies conducted in ICUs [25, 34]. Because the pH values were similar among these studies, a separate factor associated with ICU admission criteria may explain the observed differences.
  No relevant data are available in the literature on this topic in relation to OHS patients. In the present study, we assessed 189 OHS patients with a statistical power of 85 % (1 - β). No significant difference in the NIV failure rate was observed between the severe and non-severe acidosis groups, although there was a trend toward a higher rate of NIV failure in the severe acidosis sub-group (11.1 % vs. 4.3 %; *P* value = 0.083). Further studies must be performed to determine the statistical and clinical significance of these findings.
  Thus, our results are consistent with those of previous studies, suggesting that patients with AHRF secondary to COPD, ACPE, or OHS who are admitted to an RICU can be successfully treated with NIV in these units, regardless of their pH at the time of admission.
- Do patients with severe acidosis who are admitted to an RICU improve more slowly than other patients? A study of 29 COPD patients with AHRF revealed that similar durations were required for pH normalization in patients with a pH ≤ 7.25 and a pH > 7.25 and that the mean time required for both groups was 12 h [9]. In our study, the differences in the durations of pH normalization were significant, even after 24 h. In Crummy’s study [9], the clinical and functional characteristics of the patients and the site at which NIV was applied were similar to those of the present study. However, our sample was more than 20-fold larger in size, which may explain the differing results. A recent observational study conducted in ICU has revealed that ACPE patients with severe baseline hypercapnia (and acidosis) require a longer duration of NIV than those without severe baseline hypercapnia [34]. Although their study was performed in an ICU and the characteristics of their patients may have been different from those of our patients, their results are in agreement with our RICU results. No data are available on this topic in the literature with regard to OHS. Therefore, patients with AHRF and severe acidosis admitted to an RICU appear to require a longer duration of NIV treatment for pH normalization compared with those with non-severe acidosis.
  In the present study, the hospitalization durations among the patients with severe and non-severe acidosis in the three disease groups were similar. However, the RICU durations were longer for the patients with severe acidosis, especially those with COPD. In an aforementioned study evaluating COPD patients with and without severe acidosis [9], the hospitalization durations were also found to be similar between these two groups (8 days for the patients with non-severe acidosis and 9 days for those with severe acidosis). The general tendency toward a longer hospital stay observed among the patients with a DNI order was most likely associated with their older age and increased frequency of underlying comorbidities.
  Based on our data, the OHS group required less time to achieve pH normalization than the other two groups. A similar tendency was observed in another recent observational study comparing patients with COPD and OHS to those with AHRF [3]. NIV is likely to be more directly applied in OHS than in the other two diseases (ACPE and COPD). However, while NIV increased alveolar ventilation and caused respiratory muscle unloading in all three diseases, in ACPE and COPD, the final extent of improvement was more dependent on the pharmacological treatment used, such as steroids/diuretics, which can require a longer duration to achieve effects. More studies examining these issues are necessary.

Analysis of the global NIV failure rates (without dividing the patients into severe and non-severe acidotic groups) revealed that the ACPE patients experienced a higher NIV failure rate than those with the other two diseases (Fig. 1), although the rate observed in the present study (14 %) was within the published range of 4–39 % [4, 6, 11, 32, 35–37]. Other studies have also compared outcomes following NIV among patients with different diseases [11, 35–37], reporting lower [11, 37] or similar [35, 36] relative NIV failure rates in ACPE and COPD patients. These discrepancies in results may be due to the higher rates of comorbid diseases and overlap among diseases in this study (i.e., 11 patients had concomitant COPD and 14 had concomitant OHS in this study) or differences in the definitions used for NIV failure among the studies (i.e., OTI without death). Future larger studies will need to be performed to address this interesting topic.

Additional comments about the following topics are provided in Additional file 1. 1) Does acidosis improvement at intermediate time points after NIV initiation predict favorable outcomes in RICU patients? 2) Lower BMIs and higher systolic blood pressures were observed in the more acidotic COPD and OHS patients, respectively. 3) Finally, a high number of patients had a pH < 7.35 at 24 h after initiation of NIV.

### Limitations

The present large population-based study was observational because of the ethical problems associated with performing randomized trials. Patients with pneumonia and acute myocardial infarction who had an intubation order were not included, which may have affected our results.

As mentioned, the definition of NIV failure varies across studies. Hence, comparisons of our results with those of other studies should be made with caution. Some of our patients presented with associated comorbidities that were not ACPE, COPD or OHS or that overlapped with these diseases, and influences of these factors on the NIV failure rate cannot be completely excluded. The Charlson index was not found to be a predictive factor in our study.

Although the NIV failure rate did not significantly differ between the COPD group and the total population or between the DNI sub-groups, there was a tendency toward a lower NIV failure rate among the patients with severe acidosis and no DNI order. We believe that this association may be established as significant in a study of a larger sample population.

## Conclusions

In summary, our results suggest that patients with ACPE, COPD or OHS with AHRF and severe acidosis (pH ≤ 7.25) who are admitted to an RICU can be successfully treated with NIV in these units. These results may be used to determine precise RICU admission criteria. In addition, this strategy may be more cost-effective for these patients than treatment in an ICU, although further studies comparing ICU and RICU management will be required to reach a definitive conclusion on this matter.

## Abbreviations

ACPE, Acute cardiogenic pulmonary edema; AHRF, Acute hypercapnic respiratory failure; BMI, Body mass index; COPD, Chronic obstructive pulmonary disease; CPAP, Continuous positive airway pressure; DNI, Do not intubate; ICU, Intensive care unit; NIV, Noninvasive ventilation; OHS, Obesity hypoventilation syndrome; OTI, Oro-tracheal intubation; PSV, Bi-level pressure support; RICU, Respiratory intensive care units.

## Additional file

Additional file 1: Supplementary materials. (DOC 142 kb)
