# Supplementary material for: Noninvasive ventilation for severely acidotic patients in respiratory intermediate care units: Precision medicine in intermediate care units
Source: BMC Pulm Med. 2016 Jul 7;16:97. doi: 10.1186/s12890-016-0262-9 (PMC4937546; doi:10.1186/s12890-016-0262-9)

**SUPPLEMENTARY MATERIALS**

**INFORMATION ON ADDITIONAL METHODS**

**Project promotion, centers, and internal organization**

This project was initially promoted by the National Pulmonologist Society (SEPAR) through the Spanish Noninvasive Ventilation Network Organization. The Directing Committee asked Dr. Juan F Masa to develop the project, to obtain grants and to find centers with the following characteristics: 1) presence of a respiratory intermediate care unit (RICU); 2) at least three years of experience in this area; and 3) participation in at least one previous multicenter study promoted by the Spanish Noninvasive Ventilation Network Organization.

An RICU was defined as follows: a site 1) located in a specific area within a hospital integrated with or adjacent to a pulmonary area; 2) presided over by a pulmonologist; 3) with access to noninvasive monitoring procedures (e.g., electrocardiogram, oxygen saturation, transcutaneous PCO_2_, blood pressure, and respiratory rate monitoring and the monitoring of basic curves from ventilators); 4) with a nursing ratio of 1/4-6 nurses with special training in noninvasive ventilation (NIV); and 5) with 24-hour physician coverage.

Successive versions of the protocol and database variables were discussed among the researchers at three consecutive official meetings of the SEPAR and during 20 months of continuous email correspondence among the researchers. In 2010, the final versions of the protocol, database variables, and grant were made available. The coordinating center in Cáceres developed the following necessary tools with which to conduct a multicenter study: 1) electronic databases hosted on a website with a specific domain; and 2) an electronic notebook containing the study procedures (explained step-by-step), as well as the questionnaires used to standardize the research conducted among the centers. In 2011, the patient inclusion process began, and one meeting was held among the researchers following inclusion of the first 20 patients to determine whether minor changes to the protocol were necessary.

The following actions were established *a priori*: 1) monthly newsletters were sent from the principal investigator to the other researchers to report on the comparative inclusion results among the centers and to encourage the inclusion and prompt communication of results; 2) investigator meetings were established at two annual official SEPAR meetings; and 3) policy publications were developed to forecast the number of publications and authorships that would result from the study based on the numbers of patients included at the participating facilities.

**Intubation criteria**

In both groups of patients (those with a pH of less than or greater than 7.25), the following pre-defined criteria were used for intubating patients with do not intubate (DNI) order: respiratory or cardiac arrest; respiratory pauses or a heart rate <50/min with a loss of alertness or gasps for air; major agitation that was inadequately controlled by sedation; evidence of exhaustion, including active contraction of the accessory muscles, characterized by thoracic-abdominal paradoxical movement; and either massive aspiration or an inability to appropriately manage respiratory secretions, in addition to hemodynamic instability without an appropriate response to fluids and vasoactive agents.

**Ventilators**

The following ventilators were used at the included centers: BiPAP Vision and Trilogy 100 (Philips-Respironics, Murrysville, PA, USA), Elisse TM (150 and 350) and Stellar 150 (ResMed, San Diego, CA, USA), Vivo 50 (Breas Medical, Molnlycke, Sweden), Airox Supportair (Covidien-Medtronic, Minneapolis, MI, USA), Carina (Dräger, Lübeck, Germany), and Ivent 201 (Versamed Medical Systems Inc., NY, USA).

**ADDITIONAL RESULTS**

See Figure 1.

**ADDITIONAL DISCUSSION**

Is acidosis improvement at intermediate time points after the initiation of NIV a predictive factor for favorable outcomes in RICU patients? Three studies have been performed to explore this topic in chronic obstructive pulmonary disease (COPD) patients; two were performed in a conventional ward, and the other was performed in a ward with an RICU and an intensive care unit (ICU). These studies have demonstrated that acidosis is an unfavorable predictor at one [1], two [2] and four [3] hours after initiation of NIV. An additional study performed in an ICU did not find that acidosis at one hour after initiation was a predictive factor [4]. Our mean pH value was closer to that of the ICU study, although other patient characteristics were similar to those in the non-ICU studies. “Late failures”, which occur after initial improvement is observed with NIV, are frequent in ICUs (23%) [4]. Thus, one hour may have been an insufficient duration after initiation of NIV to monitor improvement in acidosis in the ICU population. We could not find similar data on this topic in the literature pertaining to acute cardiogenic pulmonary edema (ACPE) and obesity hypoventilation syndrome (OHS) patients with acute hypercapnic respiratory failure (AHRF). According to our data, among the RICU patients, pH normalization after one hour of NIV treatment was a favorable predictive factor only in the COPD group.

The COPD patients with severe acidosis had a lower body mass index (BMI), in accordance with the results of a previous study [5]. In our study and in other studies [6,7], BMI was not found to be an independent predictor of NIV failure. However, a high PaO_2_ was found to be an unfavorable predictive factor, most likely due to a high oxygen inspiratory fraction [5].

The patients with OHS and severe acidosis had a lower BMI and higher systolic blood pressure than those with non-severe acidosis. Systolic blood pressure, but not BMI, was found to be an independent predictor of NIV failure. Hypoxia and hypercapnia in an acute setting can increase sympathetic activity and lead to an increase in blood pressure. Obstructive sleep apnea is a well-known risk factor for hypertension because it increases sympathetic stimulation, and it is commonly observed in OHS. Therefore, we hypothesized that hypoxia, hypercapnia and sleep apnea may cause sympathetic stimulation and thus increased blood pressure.

A significant percentage of the patients had a pH of between 7.30 and 7.35 at 24 hours after initiation of NIV treatment, particularly those with severe baseline acidosis (Figure 2). Although we did not use acidosis as an OTI criterion, these patients experienced improvements in other clinical and respiratory functional parameters, and the majority of them achieved a favorable outcome.

**REFERENCES:**

1. Antón A, Güell R, Gómez J, Serrano J, Castellano A, Carrasco JL, Sanchis J. Predicting the result of noninvasive ventilation in severe acute exacerbations of patients with chronic airflow limitation. Chest 2000;117:828-33.
2. Confalonieri M, Garuti G, Cattaruzza MS, Osborn JF, Antonelli M, Conti G, Kodric M, Resta O, Marchese S, Gregoretti C, Rossi A. Italian noninvasive positive pressure ventilation (NPPV) study group. A chart of failure risk for noninvasive ventilation in patients with COPD exacerbation. Eur Respir J 2005;25:348-55.
3. Plant PK, Owen JL, Elliott MW. Non-invasive ventilation in acute exacerbations of chronic obstructive pulmonary disease: long-term survival and predictors of in-hospital outcome. Thorax 2001;56:708-12.
4. Moretti M, Cilione C, Tampieri A, Fracchia C, Marchioni A, Nava S. Incidence and causes of non-invasive mechanical ventilation failure after initial success. Thorax 2000;55:819-25.
5. Crummy F, Buchan C, Miller B, Toghill J, Naughton MT. The use of noninvasive mechanical ventilation in COPD with severe hypercapnic acidosis. Respir Med 2007; 101:53-61.
6. Carrillo A, Ferrer M, Gonzalez-Diaz G, Lopez-Martinez A, Llamas N, Alcazar M, Capilla L, Torres A. Noninvasive ventilation in acute hypercapnic respiratory failure caused by obesity hypoventilation syndrome and chronic obstructive pulmonary disease. Am J Respir Crit Care Med 2012;186:1279-85.
7. Ambrosino N, Foglio K, Rubini F, Clini E, Nava S, Vitacca M. Non-invasive mechanical ventilation in acute respiratory failure due to chronic obstructive pulmonary disease: correlates for success. Thorax 1995;50:755-7.

**FIGURE LEGEND**

Figure 1: The percentage of each ventilatory mode used in the patients with severe and non-severe acidosis in the three disease groups (ACPE, COPD, and OHS). PSV was used less frequently in the COPD and OHS patients with severe acidosis because they were switched to other modes, such as PSV+TV. Abbreviations: ACE=acute cardiogenic pulmonary edema, COPD=chronic obstructive pulmonary disease, OHS=obesity hypoventilation syndrome, PSV=pressure support ventilation, PSV+TV=pressure support ventilation plus target volume, PCV=pressure control ventilation, and VCV=volume control ventilation.

**Figure 1**


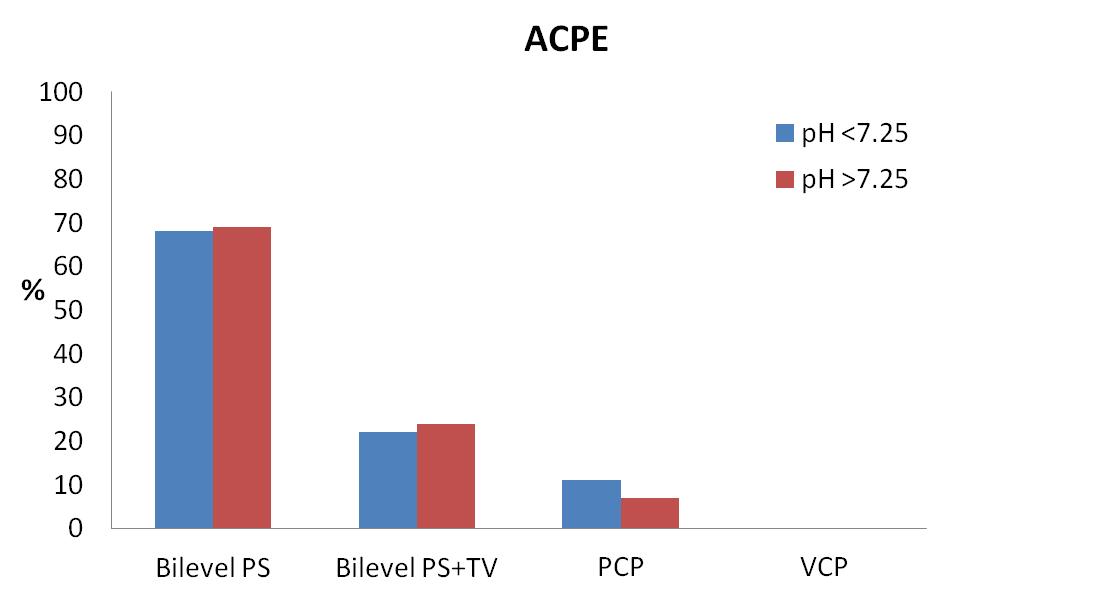


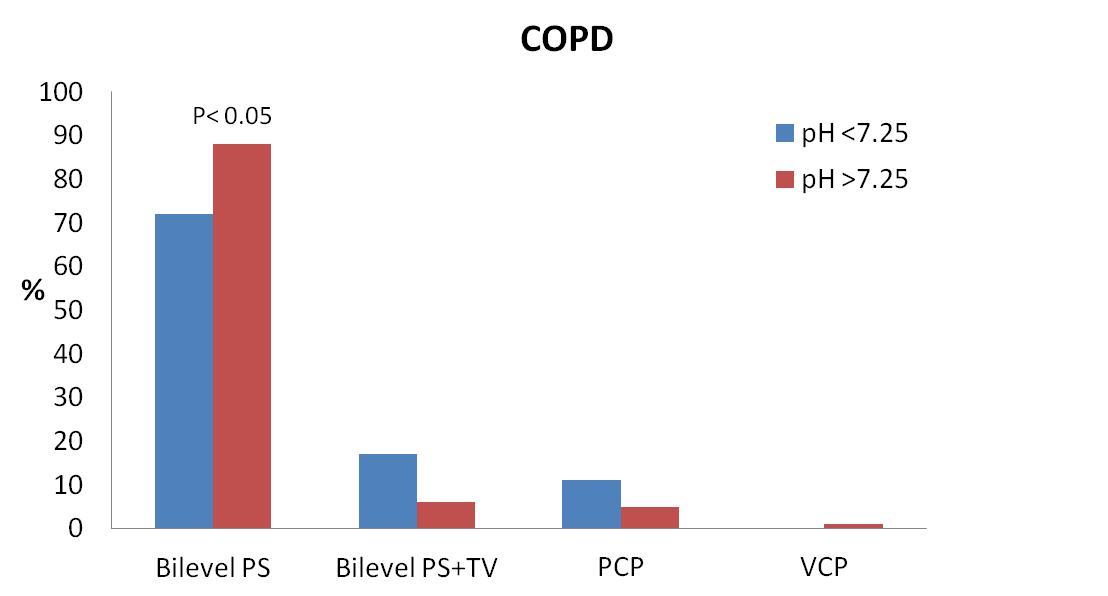


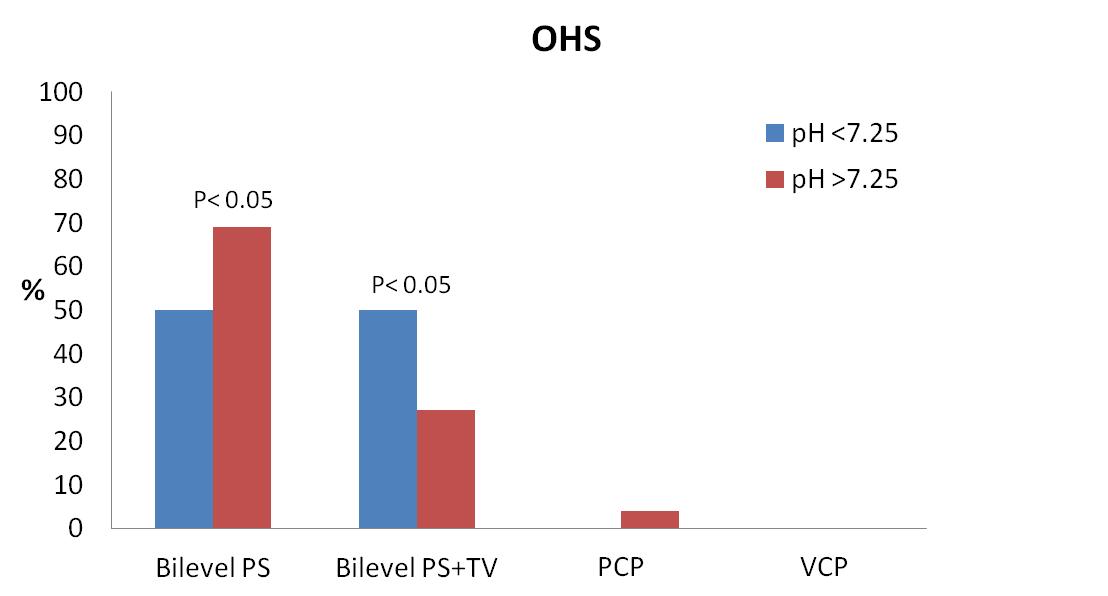

Supplement: Supplementary file 1 — Supplementary materials. (DOC 142 kb) [file 12890_2016_262_MOESM1_ESM.doc]
